# The *Li_2_* Mutation Results in Reduced Subgenome Expression Bias in Elongating Fibers of Allotetraploid Cotton (*Gossypium hirsutum* L.)

**DOI:** 10.1371/journal.pone.0090830

**Published:** 2014-03-05

**Authors:** Marina Naoumkina, Gregory Thyssen, David D. Fang, Doug J. Hinchliffe, Christopher Florane, Kathleen M. Yeater, Justin T. Page, Joshua A. Udall

**Affiliations:** 1 Cotton Fiber Bioscience Research Unit, USDA-ARS, Southern Regional Research Center, New Orleans, Louisiana, United States of America; 2 Cotton Chemistry & Utilization Research Unit, USDA-ARS, Southern Regional Research Center, New Orleans, Louisiana, United States of America; 3 USDA-ARS-Southern Plains Area, College Station, Texas, United States of America; 4 Plant and Wildlife Science Department, Brigham Young University, Provo, Utah, United States of America; Nanjing Agricultural University, China

## Abstract

Next generation sequencing (RNA-seq) technology was used to evaluate the effects of the Ligon lintless-2 (*Li_2_*) short fiber mutation on transcriptomes of both subgenomes of allotetraploid cotton (*Gossypium hirsutum* L.) as compared to its near-isogenic wild type. Sequencing was performed on 4 libraries from developing fibers of *Li_2_* mutant and wild type near-isogenic lines at the peak of elongation followed by mapping and PolyCat categorization of RNA-seq data to the reference D_5_ genome (*G. raimondii*) for homeologous gene expression analysis. The majority of homeologous genes, 83.6% according to the reference genome, were expressed during fiber elongation. Our results revealed: 1) approximately two times more genes were induced in the A_T_ subgenome comparing to the D_T_ subgenome in wild type and mutant fiber; 2) the subgenome expression bias was significantly reduced in the *Li_2_* fiber transcriptome; 3) *Li_2_* had a significantly greater effect on the D_T_ than on the A_T_ subgenome. Transcriptional regulators and cell wall homeologous genes significantly affected by the *Li_2_* mutation were reviewed in detail. This is the first report to explore the effects of a single mutation on homeologous gene expression in allotetraploid cotton. These results provide deeper insights into the evolution of allotetraploid cotton gene expression and cotton fiber development.

## Introduction

Cotton is the major source of natural fibers used in the textile industry. There are four cultivated species: AA genome diploids, *Gossypium arboretum* L. and *G. herbaceum* L.; and AADD genome allotetraploids, *G. hirsutum* L. and *G. barbadense* L. Upland cotton (*G. hirsutum*) represents about 95% of world cotton production [1]. Allotetraploid species originated around 1–2 million years ago from inter-specific hybridization between an AA-genome diploid native to Africa and Mexican DD-genome diploid [1], [2].

Cotton fibers are single-celled trichomes that emerge from the ovule epidermal cells. About 25–30% of the seed epidermal cells differentiate into spinnable fibers [3]. Fiber length ranges from short (fuzz <6 mm) to long (lint). Lint fibers of economically important *G. hirsutum* generally grow up to about 30–40 mm in length. Cotton fiber development undergoes four distinctive but overlapping stages: initiation, elongation, secondary cell wall biosynthesis, and maturation [4]. The rate and duration of each developmental stage is important to the quality attributes of the mature fiber. Cell elongation is crucial for fiber length, whereas secondary cell wall thickening is important for fiber fineness and strength.

Cotton fiber mutants are useful tools to elucidate biological processes of cotton fiber development. A cotton plant with abnormally short lint fibers was discovered in a breeding nursery of the Texas Agricultural Experiment Station in 1984. This mutant had short lint fibers (<6 mm) visually similar to those produced by Ligon lintless-1 (*Li_1_*); however, unlike the stunted and deformed vegetative morphology caused by the *Li_1_* mutation, this fiber mutant had normal vegetative growth. The trait was controlled by one dominant gene named Ligon lintless-2 (*Li_2_*) [5]. This gene was mapped to chromosome 18 (D_T_ subgenome of *G. hirsutum*) using several approaches [6]–[8]. In a fiber developmental study, Kohel *et al*. [9] observed that elongation is restricted in *Li_2_* fibers, however secondary wall development proceeds normally in proportion to fiber length. Two near-isogenic lines (NILs) of *Li_2_* with the Upland cotton variety DP5690 were developed in a backcross program at Stoneville, MS [6]. Morphological evaluation of developing fibers did not reveal apparent differences between WT and *Li_2_* NILs during initiation or early elongation up to 5 days post-anthesis (DPA). Transcript and metabolite evaluations revealed significant changes in biological processes associated with cell expansion in the *Li_2_* mutant line at peak of fiber elongation, including reactive oxygen species, hormone homeostasis, nitrogen metabolism, carbohydrate biosynthesis, cell wall biogenesis, and cytoskeleton [6], [10]. Therefore, the *Li_2_* mutation can be considered as a factor affecting cotton fiber elongation process, making it an excellent model system to study cotton fiber elongation.

In previous reports, we used microarray techniques to investigate global gene expression in *Li_2_* NILs [6], [10]. However, by using the genome sequence of *G. raimondii* [11], RNA-seq can provide a more comprehensive and accurate transcriptome analysis based on the reference DNA sequences [12]. RNA-seq offers a larger dynamic range of quantification, reduced technical variability, and higher accuracy for distinguishing and quantifying expression levels of homeologous copies than DNA microarrays [12]. Because of the limited sequence divergence between the A_T_ and D_T_ subgenomes in cotton [13], a pipeline was developed to map and categorize RNA-seq reads as originating from the A_T_ or D_T_ subgenomes [14].

In the present study we compared quantitative gene expression levels of RNA-seq data between developing fibers of *Li_2_* and its WT NILs. We investigated the *Li_2_* mutation’s effect on global transcriptional changes in subgenomes and on the functional distribution of homeologous genes during fiber elongation. These results provide deeper insights into the evolution of allotetraploid cotton gene expression.

## Results

### RNA-seq of Wild Type and *Li_2_* Developing Fibers at Peak of Elongation

Considering the cost of deep sequencing, only one time point, at the peak of elongation, was selected for RNA-seq analysis, including two biological replicates for wild type and mutant NILs. The time points 8 and 12 days post-anthesis (DPA) represent peak rates of fiber elongation. The time point 8 DPA was selected because: 1) our earlier research revealed significant transcript and metabolite changes between the *Li_2_* and wild type NILs during this time of fiber development [6], [10]; 2) the transcript level of the elongation stage-related gene *GhExp1* significantly decreased in *Li_2_* mutant fiber at 8 DPA [6].

A total of 639 million reads (each 101 bp in length) from 4 libraries were obtained by paired-end Illumina sequencing. Approximately 2.3% more reads were obtained from *Li_2_* than wild type fiber transcriptomes. From 84.4% to 90.2% of the reads were mapped to the D_5_-genome reference sequence of *G. raimondii* (Table 1). Not all the reads mapped to the reference genome sequence, probably since some of the genes were not included in the 13 large pseudo-molecules and transcripts mapped to genomic regions were outside of annotated genes. Of the mapped reads, between 29.3%–31.4% were mapped to the A_T_ subgenome and between 23.4%–25.1% were mapped to the D_T_ subgenomes of *G. hirsutum*. If the mapped reads overlapped a homeologous SNP position (SNPs between the A_T_ and D_T_ subgenomes), they were categorized as belonging to one of the two subgenomes or as a chimeric read (A-reads, D-reads, and X-reads, respectively; [14]). If a read did not overlap a homeologous SNP position, the read was unable to be categorized as originating from either the A_T_ or D_T_ subgenome (N reads; Table 1). Notably, more reads from each library were aligned to the A_T_ subgenome than to the D_T_ subgenome. Among the 37,223 genes on the 13 chromosomes of the *G. raimondii* genome, 34,692 genes (93%) had at least one mapped read from developing fibers at peak of elongation (Table 2).

**Table 1:** Results of mapping reads.

Library	*Li_2_* BR1		*Li_2_* BR2		WT BR1		WT BR2	
	count	%	count	%	count	%	count	%
**Number reads**	155,057,542	100.0	168,114,870	100.0	149,831,738	100.0	165,935,842	100.0
**A reads**	45,382,923	29.3	51,937,609	30.9	47,115,502	31.4	52,072,768	31.4
**D reads**	36,234,551	23.4	41,617,562	24.8	37,675,249	25.1	41,512,224	25.0
**X reads**	7,972,844	5.1	9,130,944	5.4	8,068,891	5.4	8,896,820	5.4
**N reads**	41,252,612	26.6	46,157,152	27.5	42,291,972	28.2	46,818,360	28.2
**Mapped total**	130,842,930	84.4	148,843,267	88.5	135,151,614	90.2	149,300,172	90.0

**Table 2:** Count of expressed genes.

Sample	*Li_2_* BR 1	*Li_2_* BR 2	*Li_2_* Total	WT BR1	WT BR2	WT Total	Total
**Expressed (≥1)**	32,413	31,845	33,553	32,170	32,481	33,460	34,692
**% of annotated genes of ** ***G. raimondii***	87.1	85.6	90.1	86.4	87.3	89.9	93.2
**Expressed (≥10)**	29,919	29,820	30,842	28,796	29,041	29,603	31,114
**% of annotated genes of ** ***G. raimondii***	80.4	80.1	82.9	77.4	78.0	79.5	83.6

### Differential Gene Expression in Developing Fibers

Counts of mapped reads were evaluated in wild type and mutant fiber transcriptomes. Genes were considered to be expressed if they had ≥10 reads mapped in one sample. Genes that were not considered to be expressed were not included in further analyses. Approximately 3% more expressed genes were detected in *Li_2_* than in wild type. Of the 37,223 genes on the 13 chromosomes of the *G. raimondii* D_5_ reference genome 29,603 (79.5%) genes were expressed in wild type and 30,842 (82.9%) genes were expressed in *Li_2_* fiber (Table 2).

Many genes had altered expression levels as a result of the *Li_2_* mutation (Table S1in File S1). Some genes were expressed in one treatment (such as *Li_2_*) but not the other treatment. For example, expressions of genes annotated as SAUR-like auxin-responsive protein (Gorai.005G257000), bHLH (Gorai.003G034700) and NAC domain transcription factor (Gorai.009G170700) were only detected in wild-type fiber. Cytokinin response factor 6 (Gorai.007G105600), UGT73C14 (Gorai.002G107900), cystein proteinase (Gorai.007G329600), MYB-like 102 (Gorai.012G132200) and WRKY transcription factor (Gorai.009G157300) were only detected in *Li_2_* fiber. The majority of these genes have not yet been functionally characterized in cotton, except of glycosyltransferase UGT73C14, which has been shown recently to be involved in ABA homeostasis [15].

**Figure 1:**
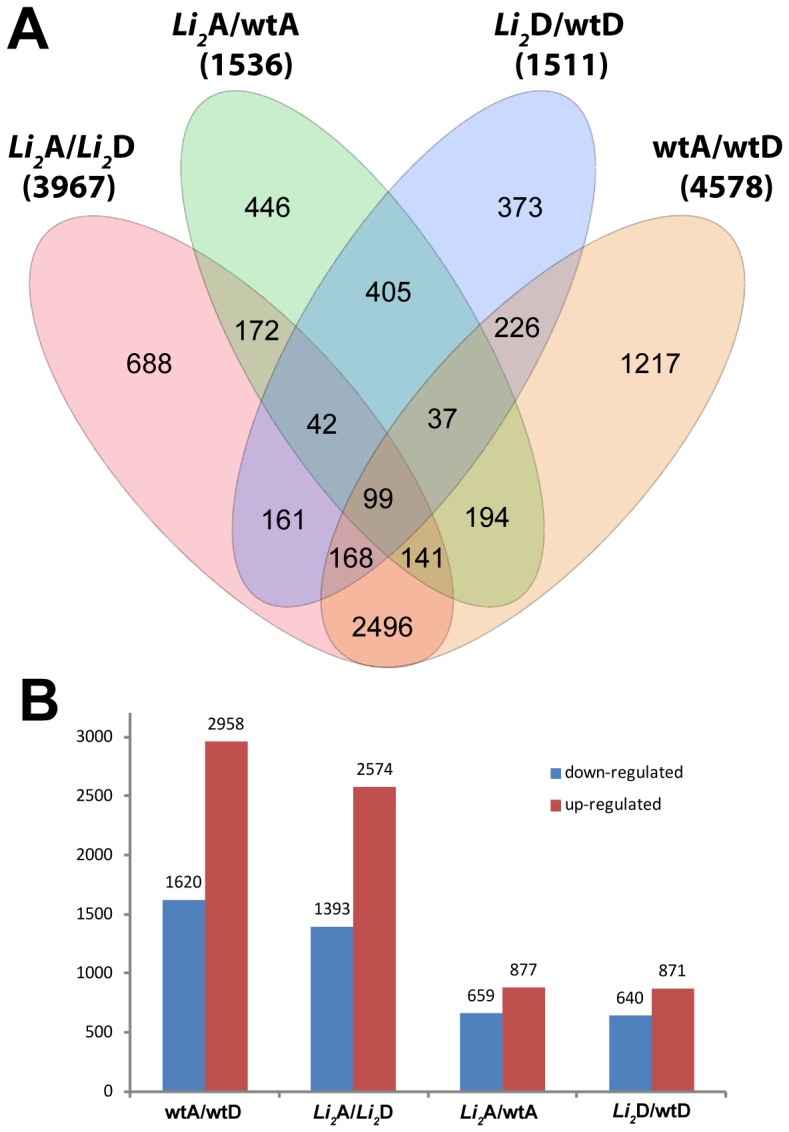
Overview of significantly regulated genes in developing fiber of *Li_2_* NILs across all comparisons. (A)Venn diagram of regulated genes in *Li_2_* versus wild type in A_T_/D_T_ subgenomes. Total number of significantly regulated genes in each comparison is indicated in parentheses. (B) The chart represents up- and down- regulated genes between subgenomes and fiber type comparisons.

The quantitative levels of mapped reads were evaluated for differential expression between elongating fibers of *Li_2_* and wild type NILs. A gene-by-gene ANOVA determined that 7,163 of 31,114 expressed genes were differentially expressed (FDR corrected *p*-value <0.05) and had ≥2-fold difference in at least one of the following comparisons: fiber type (*Li_2_* versus wild type), A_T_/D_T_ subgenomes, and combinations of these factors (statistical data for significantly regulated genes are provided in Data S1). The highest numbers of significantly differentially expressed genes were identified between the A_T_ and D_T_ subgenomes in wild type and *Li_2_*; whereas approximately 3 times fewer differentially expressed genes were detected between fiber type comparisons (Figure 1A). Of the 29,603 expressed homeologous pairs in wild type, 4,578 (wtA/wtD, 15.5%) showed significantly different expression level between subgenomes; whereas in mutant fiber of the 30,842 expressed genes, 3,967 (LiA/LiD, 12.9%) were differentially expressed between subgenomes. Therefore, the homeolog expression bias was significantly (p-value <0.0001; Chi square) reduced in *Li_2_* fiber transcriptome.

In general, the A_T_ subgenome contributed more differentially expressed genes to fiber transcriptome than did the D_T_ subgenome. Approximately two times more genes were differentially expressed in the A_T_ subgenome compared to the D_T_ subgenome in wild type (A_T_ - 2958 vs. D_T_ - 1620; Figure 1B) and mutant fiber (A_T_ - 2574 vs. D_T_ - 1393). Comparison between fiber types showed more genes were upregulated in *Li_2_* versus wild type in both subgenomes (Figure 1B). It should be noted that only about 38% (583 genes out of 1,536 in A_T_ and 1,511 in D_T_ subgenome) of significantly regulated genes between mutant and wild type overlapped between subgenomes (Figure 1A).

### Mutation Effects on Transcriptome of A_T_ and D_T_ Subgenomes of Allotetraploid *G. hirsutum*

**Figure 2:**
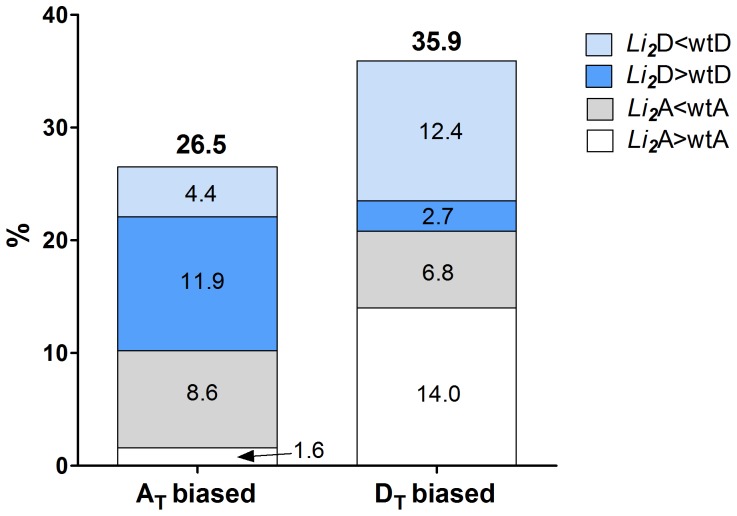
Mutation effects on A_T_ and D_T_ subgenomes of allotetraploid *G. hirsutum*. The bar chart represents percent of A_T_ and D_T_ biased genes regulated in subgenomes of *Li_2_* mutant fiber.

The effect of *Li_2_* mutation on the transcriptome of each subgenome was evaluated. The genes significantly (FDR corrected *p*-value <0.05) up-regulated (≥2-fold) in one subgenome of wild type were considered to have biased expression. Of the 2,958 A_T_ biased genes, 26.5% (784) had significantly changed the expression levels in both subgenomes of *Li_2_* as a result of mutation. However, of the 1,620 D_T_ biased genes, 35.9% (582) had significantly changed the expression levels in both subgenomes of the *Li_2_* mutant (Figure 2). Therefore *Li_2_* has a significantly greater effect (*p*-value <0.0001; Chi square) on D_T_ biased genes than A_T_ biased genes. Importantly, the majority of biased genes had significantly reduced expression levels (8.6% and 12.4%), whereas only a small portion of genes increased expression levels (1.6% and 2.7%) in the mutant. However, more genes, which were down-regulated in homeologous subgenome in wild type, had increased expression levels in the mutant fiber: 11.9% and 14% were up-regulated, whereas 4.4% and 6.8% were down-regulated (Figure 2).

**Figure 3:**
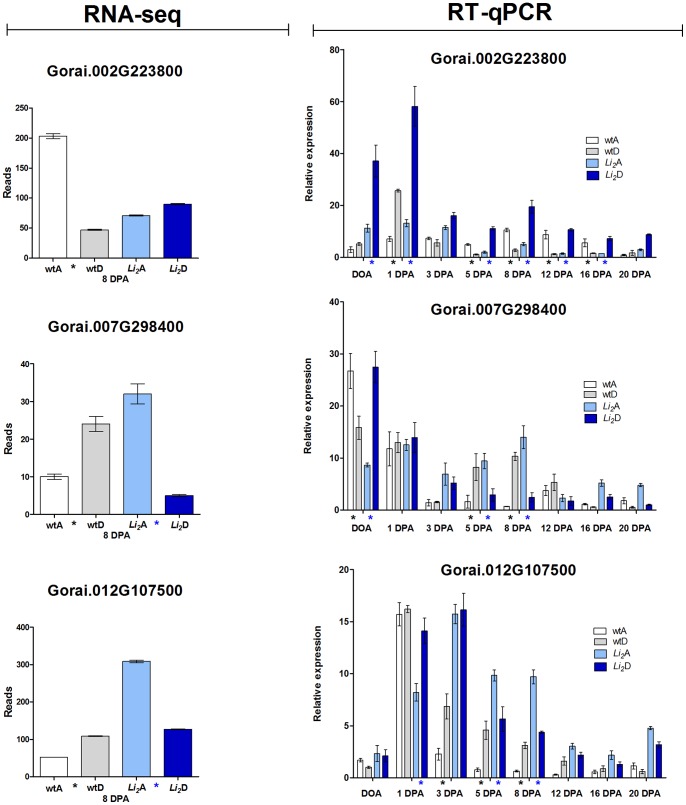
RNA-seq and rt-qPCR analysis detected reciprocal expression biases as a result of mutation. Original RNA-seq data are shown on left. Asterisks indicate significant (*p*-value <0.05) difference in gene expression level between A_T_ vs. D_T_ subgenomes in wild type (black) and mutant (blue) developing fibers. Error bars represent standard deviation from two biological replicates for RNA-seq data and three biological replicates for RT-qPCR.

Furthermore, a few homeolog pairs had reciprocal expression biases between two subgenomes as a result of mutation. Expression levels for three of these genes were tested by RT-qPCR across eight developmental time points from DOA to 20 DPA, representing initiation, elongation, and beginning of secondary cell wall biosynthesis stages (Figure 3). Interestingly, the direction of expression bias changed between developmental stages in these three genes. For example, expression of homeolog pair was biased in favor of the D_T_ subgenome for Gorai.002G223800 at initiation (1 DPA), but switched to favor the A_T_ subgenome at elongation (5–16 DPA) in wild type developing fibers, whereas expression was biased in favor of the D_T_ subgenome across all evaluated time points in mutant fibers. These results demonstrate that the *Li_2_* mutation had a greater effect on the D_T_ subgenome and also influenced direction of expression bias for some genes across developmental stages.

### Mutation Effects on Functional Distribution of Homeologous Genes during Fiber Elongation

The greater effect of *Li_2_* on D_T_ biased genes was observed in overall transcript data. In general, the subgenomes contributed unequally to different biological processes [16]; therefore diverse mutation effects could be expected on different functional categories of genes. To determine which biological processes were affected by the mutation, MapMan ontology was used (Data S2).

**Figure 4:**
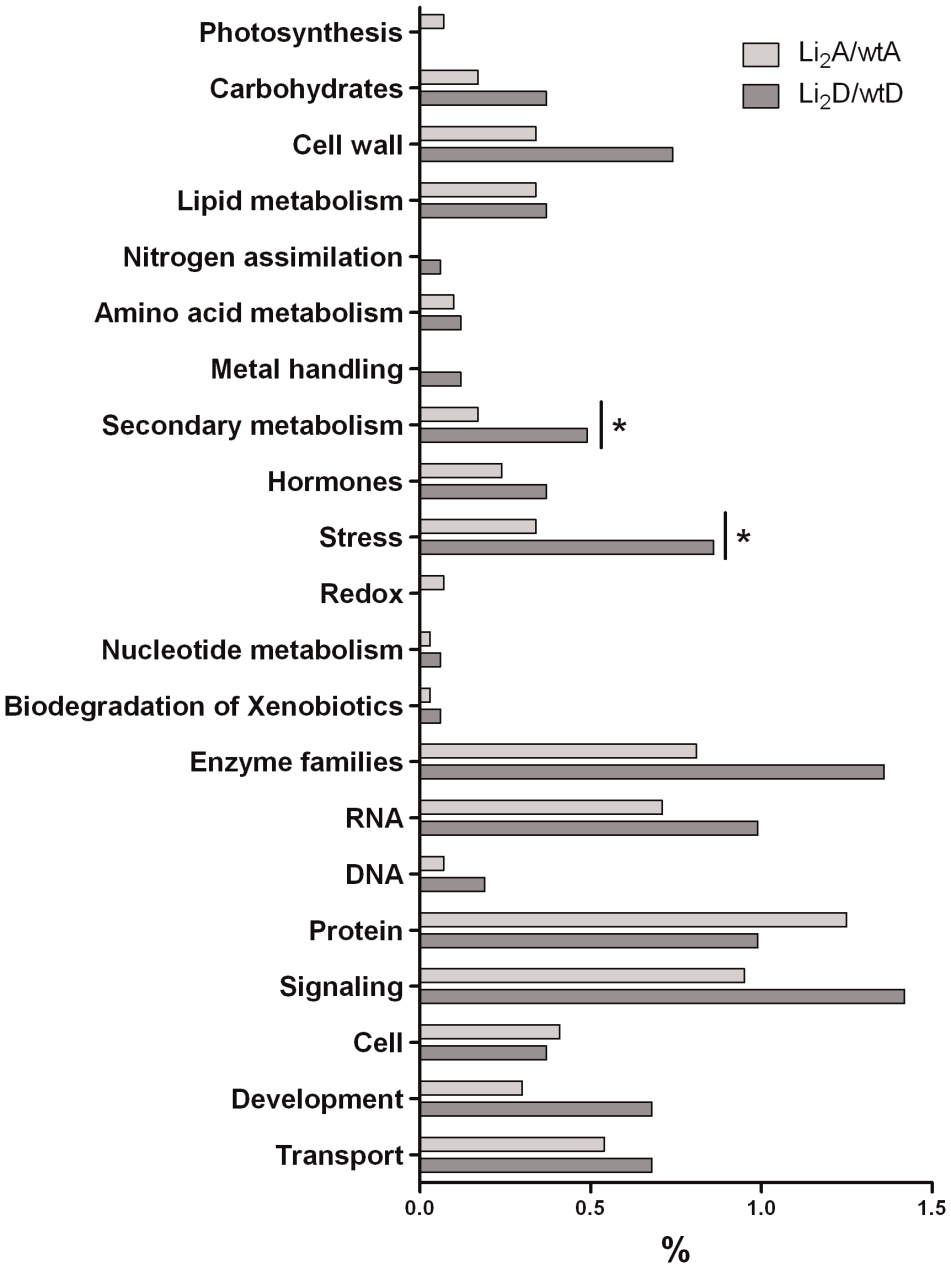
Mutation effects on functional distribution of homeolog genes during fiber elongation. Relative gene frequencies in functional categories were represented in percents from amount of biased genes in each subgenome. Asterisks indicate significant (*p*-value <0.05; Fisher’s exact test) enrichment of functional category between subgenomes with genes that changed expression in result of mutation. Table S2 in File S1 provides Fisher’s exact test results. MapMan BIN structure was used for functional categorization of genes regulated by *Li_2_* mutation. Only functional categories with more than 0.06% gene representation are shown here. Carbohydrates combine 6 BIN classes, including major and minor carbohydrates, glycolysis, fermentation, gluconeogenesis and oxidative pentose phosphate pathway.

The distribution of genes from the A_T_ and D_T_ subgenomes with significantly changed expression levels in the mutant were categorized into MapMan functional categories (Figure 4; Table S2 in File S1). Relative gene frequencies in functional categories were represented in percents of biased genes in each subgenome (2,958 A_T_ biased genes and 1,620 D_T_ biased genes). Most functional categories were biased in favor of the D_T_ subgenome with the exception of photosynthesis and redox, which only contained A_T_ homeologs. Two functional categories were significantly (*p*-value <0.05; Fisher’s exact test) biased in enrichment among D_T_ biased genes: secondary metabolism and stress (Table S2 in File S1). These results demonstrate that different biological processes were unequally affected by *Li_2_* mutation.

#### Transcriptional regulators

Transcriptional regulators (TRs) were identified in the *G. raimondii* genome based on similarity to Arabidopsis TRs and categorized into 76 families. Among them, 229 homeolog pairs were A_T_ biased and 111 were D_T_ biased in elongating cotton fibers. Of the 229 A_T_ biased TRs, 21 (9.2%) of them changed transcription level, whereas of the 111 D_T_ biased TRs 14 (12.6%) of them changed transcription level (Table 3), but this difference was statistically insignificant. Expression levels for the majority of subgenome biased homeologs decreased as the result of *Li_2_* mutation. However, six TRs (including both homeologs) had increased expression levels in mutant fibers. Three classes of TRs were the most abundant, including Aux/IAA (6 members), bHLH (5 members) and MYB (3 members). Interestingly, two of the three members of MYB TRs had increased expression due to mutation.

**Table 3:** Subgenome biased transcriptional regulators affected by mutation.

Class	Identifier	TAIR10 best hit	TAIR 10 symbol	A_T_ biased (wtA/wtD)	*Li_2_*A/wtA	D_T_ biased (wtD/wtA)	*Li_2_*D/wtD
AP2/EREBP	Gorai.006G222600	AT4G13620		**3.78**	**0.35**	**0.26**	1.47
AP2/EREBP	Gorai.007G010200	AT5G52020		**2.22**	**0.28**	**0.45**	0.68
bHLH	Gorai.007G005700	AT1G73830	BEE3	**0.14**	1.39	**7.36**	**0.37**
bHLH	Gorai.006G115500	AT2G27230	LHW	**2.71**	**2.45**	**0.37**	**2.87**
bHLH	Gorai.007G157700	AT3G07340		**5.10**	**0.50**	**0.20**	1.77
bHLH	Gorai.001G275600	AT3G26744	ICE1,SCRM	**5.82**	**0.22**	**0.17**	0.64
bHLH	Gorai.009G219100	AT4G33880	RSL2	**0.37**	1.53	**2.68**	**0.31**
C2C2(Zn) CO-like	Gorai.009G065600	AT5G24930	COL4	**2.38**	**2.17**	**0.42**	**2.23**
C2H2(Zn)	Gorai.009G092800	AT2G29660		**3.56**	**0.40**	**0.28**	0.77
C3H(Zn)	Gorai.011G126400	AT5G51980		**31.56**	**0.44**	**0.03**	1.88
CPP(Zn)	Gorai.013G121300	AT3G22780	TSO1	**0.33**	1.27	**2.99**	**0.38**
G2-like	Gorai.008G224400	AT1G32240	KAN2	**0.31**	0.93	**3.20**	**0.41**
Homeobox	Gorai.003G094500	AT3G01470	HAT5,HB-1	**0.27**	0.76	**3.76**	**0.47**
MYB	Gorai.006G195700	AT4G22680	MYB85	**12.21**	**0.31**	**0.08**	0.56
MYB	Gorai.001G015200	AT5G35550	MYB123,TT2	**3.05**	**2.13**	**0.33**	**2.08**
MYB	Gorai.007G037100	AT5G62470	MYB96	**4.72**	**2.30**	**0.21**	**5.78**
WRKY	Gorai.009G066900	AT4G31550	WRKY11	**6.87**	**0.44**	**0.15**	0.77
bZIP	Gorai.013G258300	AT3G44460	DPBF2	**0.27**	0.78	**3.68**	**0.37**
AS2, LOB	Gorai.013G016900	AT3G11090	LBD21	**0.29**	1.67	**3.48**	**0.18**
Aux/IAA	Gorai.009G132300	AT3G15540	IAA19,MSG2	**0.33**	0.97	**3.07**	**0.41**
Aux/IAA	Gorai.010G227800	AT3G15540	IAA19,MSG2	**0.10**	0.20	**9.65**	**0.21**
Aux/IAA	Gorai.001G242900	AT3G23050	AXR2,IAA7	**0.24**	**0.15**	**4.23**	**0.06**
Aux/IAA	Gorai.006G246000	AT4G14550	IAA14,SLR	**4.08**	**0.28**	**0.24**	**0.43**
Aux/IAA	Gorai.001G043900	AT4G32280	IAA29	**2.39**	**2.20**	**0.42**	**2.60**
Aux/IAA	Gorai.007G277000	AT5G43700	IAA4	**0.19**	**0.32**	**5.28**	**0.25**
General	Gorai.011G240900	AT3G52270		**3.94**	**0.40**	**0.25**	0.40
GATA type (Zn)	Gorai.005G230900	AT1G10200	WLIM1	**3.86**	**0.30**	**0.26**	0.62
TAZ domain	Gorai.007G056400	AT5G67480	BT4	**0.09**	0.99	**11.08**	**0.19**
GRP	Gorai.008G029100	AT4G39260	GRP8	**3.23**	**0.47**	**0.31**	0.74
unclassified	Gorai.013G029900	AT2G01818		**3.23**	**0.26**	**0.31**	**0.39**
unclassified	Gorai.013G258400	AT2G27580		**2.85**	**0.38**	**0.35**	0.82
unclassified	Gorai.012G146800	AT3G18870		**0.47**	1.31	**2.14**	**0.45**
unclassified	Gorai.008G221500	AT4G19050		**2.08**	**2.23**	**0.48**	**2.01**
unclassified	Gorai.010G120300	AT5G10770		**0.06**	1.17	**17.63**	**0.36**
unclassified	Gorai.004G118800	AT5G23750		**2.79**	**0.48**	**0.36**	**0.47**

The genes significantly (FDR corrected *p*-value <0.05) down-regulated or up- regulated more than 2-fold are shown in boldface and underlined.

#### Cell wall

In the cell wall functional category, 60 homeologs were A_T_ biased and 40 were D_T_ biased in fiber transcriptome. Ten (16.7%) of the A_T_ biased homeologs changed expression levels; whereas 12 (30%) of the D_T_ biased homeologs changed expression levels (Table 4), indication a higher, but statistically insignificant effect on D_T_ biased homeologs. Interestingly, more D_T_ homeologs (11) than A_T_ homeologs (4) increased transcript levels as a result of the *Li_2_* mutation. Genes encoding enzymes involved in polysaccharide degradation (14 genes) and cell wall proteins (9 genes) were the most abundant classes.

**Table 4:** Subgenome biased genes encoding enzyme involved in cell wall biosynthesis changed expression level as a result of *Li_2_* mutation.

Description	Identifier	TAIR10 best hit	A_T_ biased (wtA/wtD)	*Li_2_*A/wtA	D_T_ biased wtD/wtA	*Li_2_*D/wtD
**Hemicellulose synthesis**						
Exostosin family protein	Gorai.011G272700	AT3G45400	**4.82**	1.16	**0.21**	**2.03**
Xyloglucan β-galactosyltransferase; MUR3	Gorai.007G150100	AT2G20370	**3.36**	**0.41**	**0.30**	0.97
Xylosyltransferase; IRX9	Gorai.006G168500	AT2G37090	**0.28**	0.64	**3.63**	**0.42**
**Cell wall proteins**						
Fasciclin-like arabinogalactan 7	Gorai.001G219000	AT2G04780	**9.65**	**0.41**	**0.10**	1.72
Arabinogalactan protein 16	Gorai.007G025300	AT2G46330	**3.07**	**0.42**	**0.33**	**0.44**
Fasciclin-like arabinogalactan	Gorai.003G132100	AT3G46550	**0.05**	0.25	**20.68**	**0.43**
Fasciclin-like arabinogalactan 10	Gorai.007G092300	AT3G60900	**0.09**	2.30	**11.31**	**2.30**
Arabinogalactan protein 18	Gorai.013G048900	AT4G37450	**3.01**	0.91	**0.33**	**2.51**
Fasciclin-like arabinogalactan 17	Gorai.006G147500	AT5G06390	**2.08**	**0.42**	**0.48**	0.59
Fasciclin-like arabinogalactan 1	Gorai.013G152900	AT5G55730	**3.71**	**2.62**	**0.27**	1.65
Proline-rich protein 2	Gorai.002G193500	AT2G21140	**0.23**	0.89	**4.35**	**0.37**
Reversibly glycosylated polypeptide 1	Gorai.001G090200	AT3G02230	**0.33**	**2.04**	**3.07**	1.99
**Degradation**						
Peptidoglycan-binding LysM domain-containing protein	Gorai.008G281400	AT5G62150	**19.29**	**0.35**	**0.05**	1.68
Glycosyl hydrolase 9B8	Gorai.007G170600	AT2G32990	**0.10**	1.57	**10.48**	**0.37**
Glycosyl hydrolase	Gorai.006G118100	AT5G20950	**0.17**	1.33	**6.02**	**0.49**
α-L-fucosidase 1	Gorai.008G282700	AT2G28100	**4.69**	0.86	**0.21**	**2.36**
β-Xylosidase 1	Gorai.011G198200	AT5G49360	**2.01**	1.88	**0.50**	**2.25**
β-Xylosidase 2	Gorai.008G140600	AT1G02640	**0.31**	0.71	**3.18**	**0.22**
Rhamnogalacturonate lyase	Gorai.002G138200	AT1G09890	**3.63**	1.60	**0.28**	**3.51**
Rhamnogalacturonate lyase	Gorai.007G231600	AT2G22620	**0.40**	2.06	**2.53**	**2.33**
Rhamnogalacturonate lyase	Gorai.008G089300	AT2G22620	**11.16**	**0.20**	**0.09**	**6.32**
Polygalacturonase 2	Gorai.002G161400	AT1G70370	**0.35**	**0.46**	**2.85**	**0.38**
Polygalacturonase 2	Gorai.005G136300	AT1G70370	**5.03**	**0.39**	**0.20**	1.55
Pectin lyase-like	Gorai.004G138900	AT4G23500	**0.36**	1.48	**2.81**	**2.50**
Pectin methylesterase 1	Gorai.009G147800	AT1G53840	**11.39**	**2.27**	**0.09**	3.18
Pectinacetylesterase	Gorai.004G176200	AT4G19410	**0.38**	**2.11**	**2.66**	1.89
**Modification**						
Expansin A4	Gorai.003G131000	AT2G39700	**2.97**	0.51	**0.34**	**0.48**
Expansin A4	Gorai.007G376300	AT2G39700	**2.07**	1.66	**0.48**	**2.27**
Expansin A4	Gorai.012G104400	AT2G39700	**2.10**	1.56	**0.48**	**2.50**
Expansin A8	Gorai.012G014400	AT2G40610	**0.24**	**0.32**	**4.11**	**0.23**
Xyloglucan endotransglucosylase/hydrolase	Gorai.004G030500	AT4G25810	**4.32**	**0.40**	**0.23**	0.43
Xyloglucan endotransglucosylase/hydrolase, GhXTH1	Gorai.007G057400	AT4G37800	**0.16**	0.97	**6.06**	**0.41**

The genes significantly (FDR corrected *p*-value <0.05) down-regulated or up- regulated more than 2-fold are shown in boldface and underlined.

### Validation of Illumina RNA-seq Expression and Subgenome Specific Categorization of Reads by RT-qPCR Analysis

To test the reliability of Illumina sequencing and SNP-based categorization of reads to the A_T_ or D_T_ subgenome of allotetraploid *G. hirsutum*, RT-qPCR analysis was performed for a subset of 8 genes (selected from Table S1 in File S1) expressed only in WT or *Li_2_* NILs, and for a subset of 11 genes (selected from Tables 3 and 4) that showed subgenome biased expression. Overall, the results of RT-qPCR analysis were consistent with results of RNA-seq analysis for 19 selected genes (Figures S1 and S2). RT-qPCR analysis confirmed silencing or activation of the expression by the *Li_2_* mutation for the subset of 8 genes (Figure S1). Correlation analysis of the expression patterns revealed strong correlations between RNA-seq and RT-qPCR data. In the subset of 11subgenome biased genes, 7 genes showed 100% correlation (*p*-value <0.05) and 4 genes showed 99% correlation (*p*-value >0.05; Figure S2).

## Discussion

Our results demonstrate that the A_T_ subgenome in general contributed approximately two times more significantly induced genes to the fiber transcriptome than the D_T_ subgenome; however, the *Li_2_* mutation had greater effects on the D_T_ subgenome than the A_T_ subgenome.

### Global Transcript Changes in Subgenomes of *G. hirsutum* Following *Li_2_* Mutation

The role of the A_T_ and D_T_ subgenomes in determination of fiber quality in allotetraploid cotton has been extensively discussed in the literature. Allopolyploidization resulted in significant improvements in the desirable agronomic fiber traits in the allotetraploid species in comparison with the diploid progenitors [17], [18]. The first evidences showing that QTLs for fiber quality (including length, strength and fineness) were associated with DNA markers mapped to the D_T_ subgenome rather than the A_T_ subgenome was published by Paterson’s group [18]. Review of numerous QTLs published from 1998 to 2007 confirmed the observation that the D_T_ subgenome plays a larger role in genetic control of fiber traits [19]. A microarray study published by Wendel’s group found that the homeolog expression in *G. hirsutum* was biased in favor of the D_T_ subgenome in fiber cells [16]. Similar results were reported by Lacape and coauthors utilizing deep sequencing approach to analyze the fiber transcriptome of two allotetraploid species *G. hirsutum* and *G. barbadense* [20]. From an evolutionary point of view, these observations are surprising since the genes responsible for improved fiber properties evolved in the diploid AA genome before polyploidization [21]. None of the DD genome species produce spinnable fibers [22].

There are discrepancies in the literature regarding homeolog bias in contribution to fiber traits. Using a core set of 111 RFLP markers, Ulloa and coauthors revealed that the A_T_ subgenome exhibited 68% of QTLs from the five chromosomes, whereas the D_T_ subgenome exhibited only 32% of QTLs from the three chromosomes [23]. Another study utilizing combinations of markers found more fiber trait QTLs in the A_T_ subgenome than in the D_T_ subgenome [24]. The expression analysis of ESTs derived from immature ovules of *G. hirsutum* TM-1 revealed significant enrichment in all functional categories for A_T_ subgenome ESTs [25].

These inconsistencies could be explained by technical limitations. The QTL studies reported in the current literature are detecting only a small subset of the genes related to fiber traits that may not cover the whole genome and could be insufficient to conclude which subgenome more significantly contributes to fiber properties [19]. The microarray studies evaluated a limited number of homeologous gene pairs, resulting in limited statistical power [16], [26]. Lacape and coauthors used next generation DNA sequencing technology for fiber transcriptome analysis; however, they evaluated only 617,000 good quality reads from four libraries without biological replication [20]. Unlike previous studies we obtained ∼160 million reads per sample for each of two biological replicates (Table 1), providing ∼5.6 times coverage of the *G. hirsutum* genome (∼2.83 Gb per haploid [27]), which is more than enough to deliver statistically powerful transcriptional analysis. Our observation of higher expression of A_T_ than D_T_ genes in the fiber transcriptome is consistent with the results of cotton ovules ESTs analysis [25] and reflects the evolutionary role of the AA diploid progenitor in fiber quality traits of allotetraploid cotton.

It is interesting to note that the *Li_2_* mutation coincides with an increase in the number of expressed genes, but the homeolog expression bias was significantly decreased in *Li_2_* fiber. How expression of homeologous genes is regulated in polyploids is still unclear, although it could involve altered regulatory interactions and rapid genetic and epigenetic changes in subgenomes [28]. The evolution homeolog-specific expression after polyploidization has been extensively studied in allotetraploid cotton. Higher rates of homeolog expression bias in natural allotetraploids than in hybrid and synthetic polyploid cottons suggested that the extent of homeolog expression bias increases over time from hybridization through evolution [26], [29], [30]. The *Li_2_* mutation is negative for desirable fiber quality traits, resulting in extremely short lint fiber. Significant reduction of homeolog expression bias in short fiber suggests that the extent of homeolog expression bias is also important for fiber quality characteristics.

We observed a reciprocal switch for some genes in expression bias between homeologs during fiber developmental stages in the mutant. A high degree of expression differences between homeologous genes that are developmentally and stress regulated was reported in cotton [17], [31], [32]. A high-resolution genome-specific study of expression profiling for 63 gene pairs in 24 tissues in allopolyploid and their diploid progenitor cotton species demonstrated that the majority of expression differences between homeologs are caused by *cis*-regulatory divergence between the diploid progenitors; however, some degree of transcriptional neofunctionalization was detected as well [32].

The *Li_2_* mutation was mapped to the D_T_ subgenome [6]–[8]; however, the mutated gene and the nature of mutation are currently unknown. The greater mutation effect on the D_T_ than on the A_T_ subgenome observed here suggests two possible mechanisms. The network of regulatory interactions may have been interrupted by a mutation in the D_T_ subgenome resulting in transactivation or repression of individual gene expression levels and expression cascades. Alternatively an epigenetic modulation may preferentially target the D_T_ subgenome. It has been shown that small RNAs can control gene expression and epigenetic regulation in response to hybridization [33]–[36]. For example, miRNAs in allopolyploid Arabidopsis triggered unequal degradation of parental target genes [33]. Similarly, in rice hybrids small RNA populations inherited from parents were responsible for biased expression [36]. Additional investigations of epigenetic and chromatin level modifications will provide insights into causes of gene expression variation between subgenomes.

### TRs and Cell Wall Functional Categories of Genes Regulated by *Li_2_* Mutation

Previous transcriptomics and metabolomics studies have shown that the *Li_2_* mutation terminated the cotton fiber elongation process [6], [10]; therefore, genes with changed expression level in the mutant could be involved in elongation. In the present work, we described in detail TRs and cell wall functional categories, which are critical for fiber developmental processes. Many genes in this list (Table 3 and Table 4) were not functionally characterized in cotton; although, based on sequence similarity to genes characterized in Arabidopsis, they could be involved with fiber elongation and represent candidates for further functional analysis in cotton.

#### Transcriptional regulators

Many TRs regulated by *Li_2_* mutation are involved in hormonal signaling and development. Particularly, two AP2/EREBPs and six Aux/IAAs were in the pool of TRs affected by *Li_2_* mutation (Table 3). Plant hormones are important for fiber development. It is well documented that exogenous applications of auxins and gibberellic acid stimulate the differentiation of fibers and promote elongation, while abscisic acid and cytokinins inhibit fiber growth in an *in vitro* cotton ovule culture system [37], [38]. Among the auxin responsive genes, Gorai.009G132300 and Gorai.010G227800, whose transcript abundances were significantly reduced in the D_T_ genome of *Li_2_*, showed sequence similarity to grapevine VvIAA19 regulator [39]. Transgenic Arabidopsis plants over expressing VvIAA19 exhibited faster growth, including root elongation and floral transition, than the control, suggesting that grape Aux/IAA19 protein is likely to play a crucial role as a plant growth regulator. In the group of bHLH family of TRs, Gorai.007G005700 transcript abundance was significantly reduced in the D_T_ genome of *Li_2_* and showed sequence similarity to Arabidopsis *BEE3*, one of several redundant positive regulators of brassinosteroids signaling required for normal growth and development [40].

The actin cytoskeleton plays an important role in cell morphogenesis; down-regulation of *GhACT1* disrupted the actin cytoskeleton network in fibers that resulted in inhibition of fiber elongation [41]. A GATA type TR Gorai.005G230900, a homolog of Arabidopsis *WLIM1*, was down-regulated in the D_T_ subgenome of *Li_2_*; a recent study revealed that plant LIM-domain containing proteins (LIMs) define a highly specialized actin binding protein family, which contributes to the regulation of actin bundling in virtually all plant cells [42].

#### Cell wall

The plant cell wall has a dual role during elongation: to sustain the large mechanical forces caused by cell turgor and to permit controlled polymer extension generating more space for protoplast enlargement [43]. The active biosynthesis of matrix polysaccharides along with increased activity of cell wall loosening enzymes has been considered to be associated with cell wall extension [44]–[48]. Expression levels of genes encoding enzymes involved in xyloglucan and glucuronoxylan biosynthesis were decreased as a result of *Li_2_* mutation. Particularly, xyloglucan β-galactosyltransferase (Arabidopsis homolog, *MUR3* [49]) and xylosyltransferase (*IRX9* [50]) were down-regulated in the A_T_ or D_T_ subgenomes of mutant fibers (Table 4).

Among cell wall proteins arabinogalactans were the most abundant members. Arabinogalactan-proteins have been implicated in many processes involved in plant growth and development, including cell expansion [51], [52].

Primary cell wall expansibility and strength is in part mediated by a group of enzymes that comprise a large family of cell wall modifying proteins, the xyloglucan endotransglycosylase/hydrolases (XTHs). XTHs are apoplast-localized enzymes that cleave and reattach xyloglucan polymers [53], [54]. The role of XTHs in cotton fiber elongation has been demonstrated: transgenic over-expression of *GhXTH1* in cotton increased fiber length up to 20% [55]. D_T_ biased Gorai.007G057400 corresponding to *GhXTH1* was down-regulated in mutant fiber.

## Conclusion

Repeated polyploidization over evolutionary time has played a significant role in adding genetic variation to the genomes of plant species. The evolution of the homeolog expression after polyploidization has been extensively studied in cotton comparing expression profiling between parental diploids and natural and synthetic allopolyploid species. This is the first report that explored the effects of a single mutation on the homeolog expression of allotetraploid cotton. Our results showed that significant reduction of the homeolog expression bias in mutant fiber correlates with negative fiber traits, indicating that the extent of homeolog expression bias is important for fiber quality characteristics. In addition, we observed significantly greater mutation effects on the D_T_ than on the A_T_ subgenome that might be explained by localization of the mutated gene. Additional studies using numerous naturally occurring cotton fiber mutations are needed to confirm these observations. This work will lead to an understanding of how gene regulation between A_T_ and D_T_ homeologs contributes to enhanced fiber morphology in cultivated cotton allopolyploids.

## Materials and Methods

### Plant Material and RNA Isolation

The cotton short fiber mutant *Li_2_* was developed as a near-isogenic line (NIL) with the WT upland cotton line DP5690 as described before [6]. Growth conditions and fiber sampling were previously described [6]. Cotton bolls were harvested at the following time-points during development: day of anthesis (DOA), 1, 3, 5, 8, 12, 16, and 20 days post-anthesis (DPA). Cotton fibers were isolated from developing ovules using a glass bead shearing technique to separate fibers from the ovules [56]. Total RNA was isolated from detached fibers using the Sigma Spectrum Plant Total RNA Kit (Sigma-Aldrich, St. Louis, MO) with the optional on column DNase1 digestion according to the manufacturer’s protocol. The concentration of each RNA sample was determined using a NanoDrop 2000 spectrophotometer (NanoDrop Technologies Inc., Wilmington, DE). The RNA quality for each sample was determined by RNA integrity number (RIN) using an Agilent Bioanalyzer 2100 and the RNA 6000 Nano Kit Chip (Agilent Technologies Inc., Santa Clara, CA) with 250 ng of total RNA per sample.

### RT-qPCR Analysis

The experimental procedures and data analysis related to RT-qPCR were performed according to the Minimum Information for Publication of Quantitative Real-Time PCR Experiments (MIQE) guidelines [57]. Eight fiber developmental time-points mentioned above were used for RT-qPCR analyses of homeolog pairs which showed reciprocal expression biases. Only one time point, 8 DPA, was used for RT-qPCR confirmation of RNA-seq data of selected genes. The detailed description of reverse transcription, qPCR and calculation were previously reported [6]. Single nucleotide polymorphisms that distinguish the A_T_ and D_T_ subgenome copies of the selected genes were identified by aligning reads from the RNA-seq data to the *G. raimondii* reference mRNA sequences [11]. These homeologous SNPs were used to design subgenome specific primers by the SNAPER approach, whereby an additional mismatch is included near the end of the SNP-specific primers to increase stringency [58]. Primer sequences are provided in Table S3 and Table S4 in File S1. Correlations of biased expression patterns between RNA-seq and RT-qPCR data were calculated using GraphPad Prism 5 software (Pearson test).

### Library Preparation and Sequencing

RNA samples from *Li_2_* and wild type cotton fiber at 8 DPA (in two biological replicates) were subjected to paired-end Illumina mRNA sequencing (RNA-seq). Library preparation and sequencing were conducted by Data2Bio LLC (2079 Roy J. Carver Co-Laboratory, Ames, Iowa). Indexed libraries were prepared using the Illumina protocol outlined in the TruSeq RNA Sample Prep Guide (Part# 15008136 Rev. A, November 2010). The library size and concentration were determined using an Agilent Bioanalyzer. The indexed libraries were combined and seeded onto one lane of the flowcell. The libraries were sequenced using 101cycles of chemistry and imaging, resulting in paired end (PE) sequencing reads with length of 2×101 bp. The raw reads were submitted to the Sequence Read Archive (accession number SRP026301).

### Processing of Illumina RNA-Seq Reads and Mapping to A_T_ and D_T_ Subgenomes of *Gossypium hirsutum*

The reads were trimmed with SICKLE (https://github.com/najoshi/sickle) using a quality score cutoff of 20. Mapping the reads (in pairs where both reads of a pair passed trimming) to the 13 chromosomes of the *G. raimondii* genome D_5_ v2 reference sequence was performed using GSNAP [59]. Default parameters were used, but with the flags “-n 1 -Q” which means that only a single mapping was reported for each read, and reads with multiple equally good hits were thrown away rather than randomly mapped. We used a cotton SNP index generated between DD genome *G. raimondii* and the AA genome *G. arboreum* to categorize reads of the allotetraploid *G. hirsutum* as belonging to the A_T_ or D_T_ subgenomes according to the method reported previously [14].

### Digital Gene Expression Analysis

The comparison of the number of reads mapped to the genes of *G. raimondii* reference genome was used as an indicator of the relative digital gene expression (DGE). The JMP/Genomics 6.0 (SAS, Cary, NC, USA) was used for data normalization and statistical analysis. The data was normalized using TMM (Trimmed Mean of M component) method [60]. Genes with less than 10 reads in one sample were removed before normalization; from 37,223 genes assigned to chromosomes, 31,114 genes passed filtering conditions and were processed for normalization. The ANOVA process was fit to the normalized data, with the data following a Poisson distribution. This was accomplished with a generalized linear mixed model for each gene: ***Y_ij_*** **=** ***T_i_*** **+** ***G_j_*** **+** ***TG_ij_*** **+** ***E_ijk_***, where ***T*** is the treatment effect for the *i*th biological treatment (*Li_2_* or wild-type fiber), ***G*** is the specific subgenome type effect for the *j*th subgenome type (A_T_, D_T_, X and N categorized reads), their interaction (***TG***), and the error term (***E***). The linear model was used to test the null hypothesis that expression of a given gene was not different. Specifically, multiple comparisons were made between fiber type (*Li_2_* versus wild type) and A_T_/D_T_ subgenomes as well as combinations of these factors, such as fiber type in A_T_ and D_T_ subgenomes. We identified genes for which the difference in expression levels within these *a priori* questions were significantly different (false discovery rate≤0.05) [61].

### Functional Categorization of Genes

Functional categorization of genes was performed using MapMan ontology [62]; the MapMan mapping for *G. raimondii* is available at http://mapman.gabipd.org/. Fisher’s exact test was used to estimate enrichment or depletion relative to background of functional categories with differentially regulated genes.

## Supporting Information

Figure S1 **RT-qPCR confirmation of silencing or activation of genes as a result of mutation.** Bar charts represent RNA-seq and RT-qPCR data (side by side) at 8 DPA of fiber development for 8 randomly selected genes from Table S1 in File S1. Error bars indicate standard deviation from two biological replicates for RNA-seq data and three biological replicates for RT-qPCR.
(TIF)

Figure S2 **RT-qPCR confirmation of biased expression of homeolog pairs.** Bar charts represent RNA-seq and RT-qPCR data (side by side) at 8 DPA of fiber development for 11 randomly selected genes from Table 3 and Table 4. Pearson correlation (GraphPad Prism 5 software) of expression patterns for selected genes between RNA-seq and RT-qPCR data is provided in the table; correlation coefficients with p-value less than 0.05 are shown in boldface and underlined. Error bars indicate standard deviation from two biological replicates for RNA-seq data and three biological replicates for RT-qPCR.
(TIF)

File S1 **Supporting tables.** **Table S1.** Silencing or activation of genes as a result of mutation. **Table S2.** Mutation effects on functional distribution of homeolog genes. Fisher’s exact test results. **Table S3.** Primer’s sequences for detection expression of homeolog pairs. **Table S4.** Primer’s sequences.
(DOCX)

Data S1 **Statistical data for significantly regulated genes.**
(TXT)

Data S2 **A_T_/D_T_ biased genes annotated by MapMan ontology.**
(TXT)
