# Supplementary figures and images for: The Li2 Mutation Results in Reduced Subgenome Expression Bias in Elongating Fibers of Allotetraploid Cotton (Gossypium hirsutum L.)
Source: PLoS One. 2014 Mar 5;9(3):e90830. doi: 10.1371/journal.pone.0090830 (PMC3944810; doi:10.1371/journal.pone.0090830)

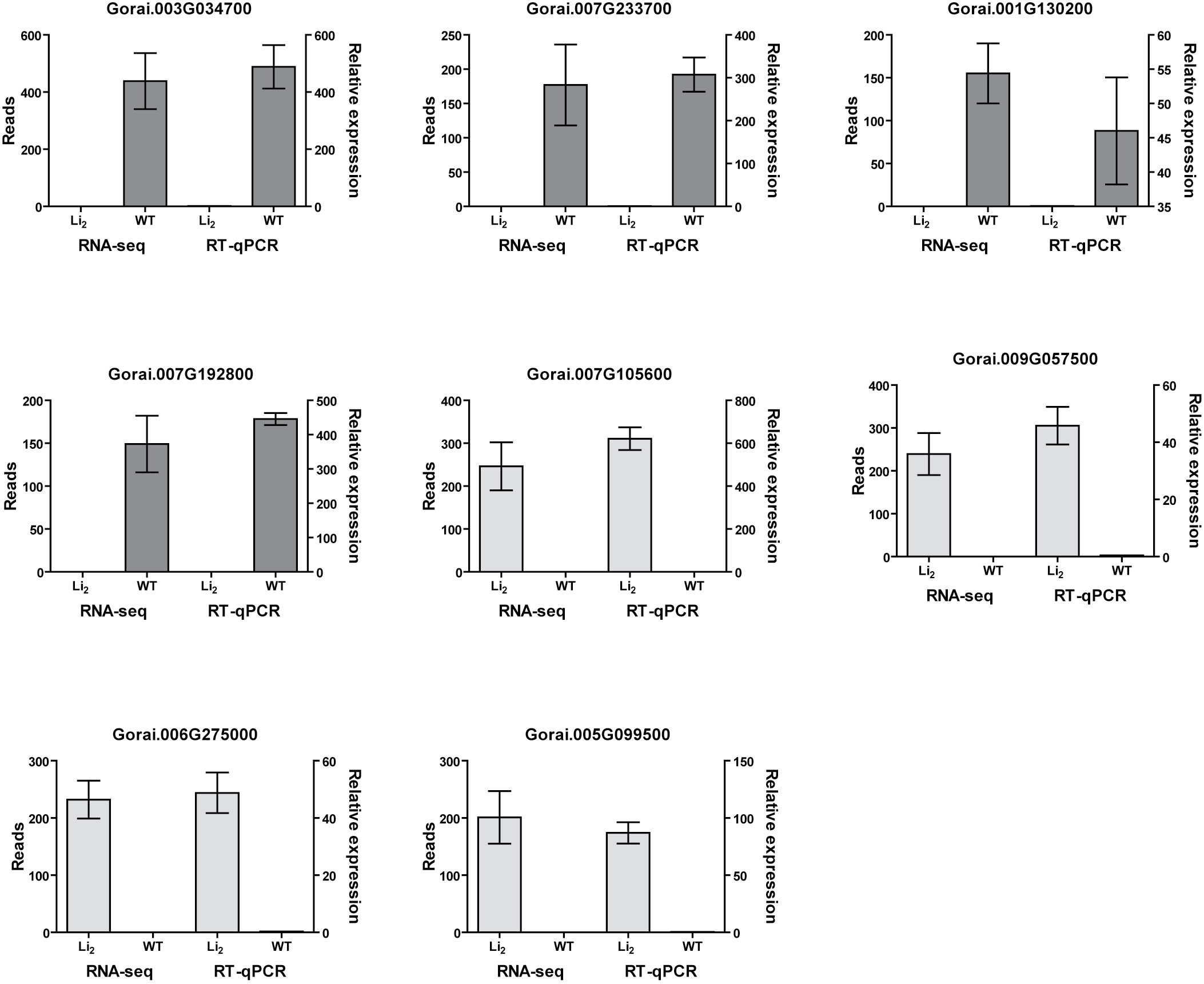

Supplement: Figure S1 — RT-qPCR confirmation of silencing or activation of genes as a result of mutation. Bar charts represent RNA-seq and RT-qPCR data (side by side) at 8 DPA of fiber development for 8 randomly selected genes from Table S1 in File S1. Error bars indicate standard deviation from two biological replicates for RNA-seq data and three biological replicates for RT-qPCR. (TIF) [file pone.0090830.s001.tif]

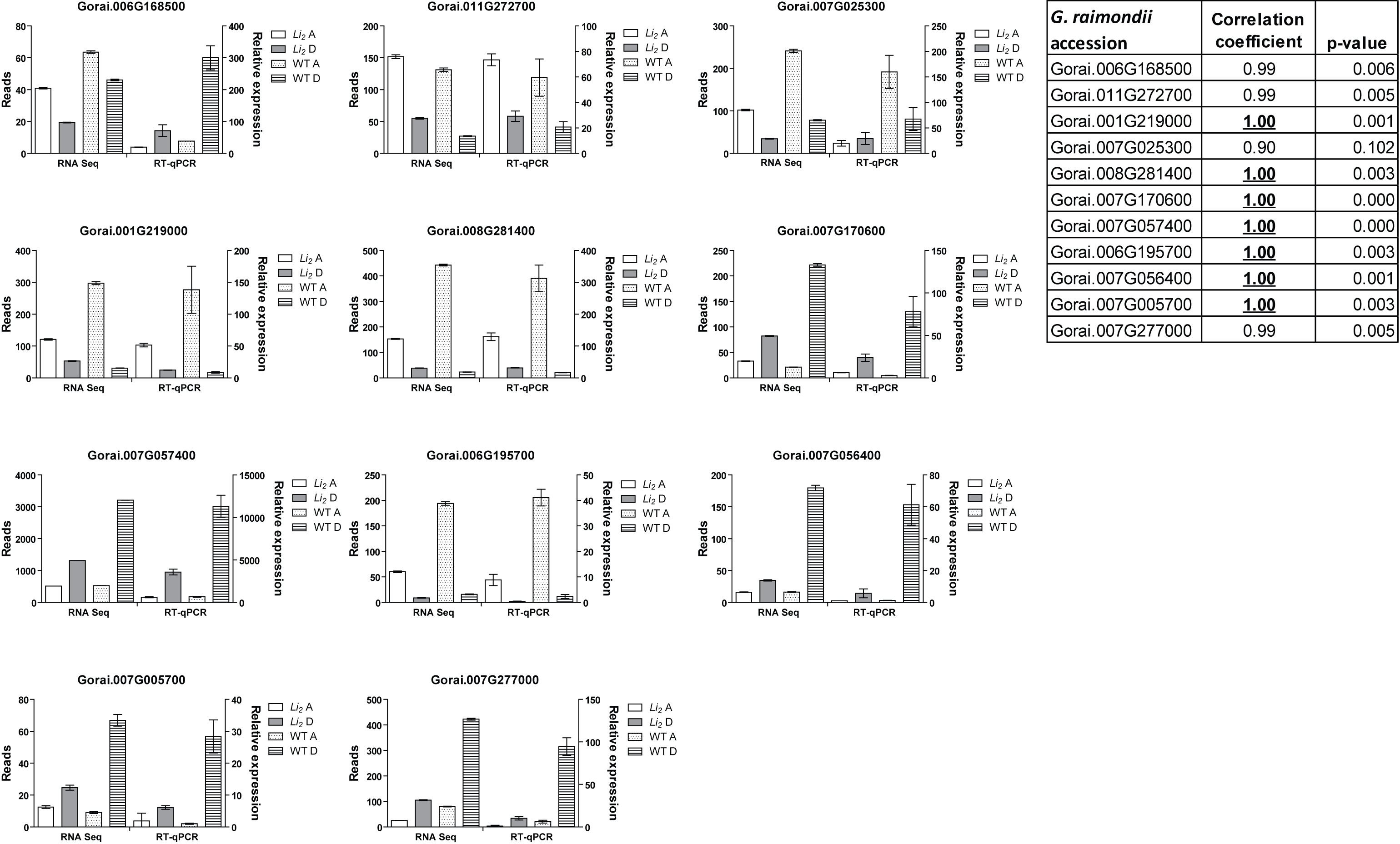

Supplement: Figure S2 — RT-qPCR confirmation of biased expression of homeolog pairs. Bar charts represent RNA-seq and RT-qPCR data (side by side) at 8 DPA of fiber development for 11 randomly selected genes from Table 3 and Table 4. Pearson correlation (GraphPad Prism 5 software) of expression patterns for selected genes between RNA-seq and RT-qPCR data is provided in the table; correlation coefficients with p-value less than 0.05 are shown in boldface and underlined. Error bars indicate standard deviation from two biological replicates for RNA-seq data and three biological replicates for RT-qPCR. (TIF) [file pone.0090830.s002.tif]
