# Supplementary material for: The Li2 Mutation Results in Reduced Subgenome Expression Bias in Elongating Fibers of Allotetraploid Cotton (Gossypium hirsutum L.)
Source: PLoS One. 2014 Mar 5;9(3):e90830. doi: 10.1371/journal.pone.0090830 (PMC3944810; doi:10.1371/journal.pone.0090830)
Supplement: File S1 — Supporting tables. Table S1. Silencing or activation of genes as a result of mutation. Table S2. Mutation effects on functional distribution of homeolog genes. Fisher’s exact test results. Table S3. Primer’s sequences for detection expression of homeolog pairs. Table S4. Primer’s sequences. (DOCX) [file pone.0090830.s003.docx]

**The *Li_2_* mutation results in reduced subgenome expression bias in elongating fibers of allotetraploid cotton (*Gossypium hirsutum* L.)**

Marina Naoumkina^1^*, Gregory Thyssen^1^, David D. Fang^1^, Doug J Hinchliffe^2^, Christopher Florane^1^, Kathleen M. Yeater^3^, Justin T. Page^4^ and Joshua A. Udall^4^

^1^Cotton Fiber Bioscience Research Unit, USDA-ARS, Southern Regional Research Center, New Orleans, LA, USA.

^2^Cotton Chemistry & Utilization Research Unit, USDA-ARS, Southern Regional Research Center, New Orleans, LA, USA.

^3^ USDA-ARS-Southern Plains Area, College Station, TX, USA.

^4^Plant and Wildlife Science Department, Brigham Young University, Provo, UT, USA.

* Correspondence: [marina.naoumkina@ars.usda.gov](mailto:marina.naoumkina@ars.usda.gov); telephone (504)-286-4262; fax (504)-286-4250

[gregory.thyssen@ars.usda.gov](mailto:gregory.thyssen@ars.usda.gov)

[david.fang@ars.usda.gov](mailto:david.fang@ars.usda.gov)

[doug.hinchliffe@ars.usda.gov](mailto:doug.hinchliffe@ars.usda.gov)

[chris.florane@ars.usda.gov](mailto:chris.florane@ars.usda.gov)

[kathleen.yeater@ars.usda.gov](mailto:kathleen.yeater@ars.usda.gov)

[jtpage68@gmail.com](mailto:jtpage68@gmail.com)

[jaudall@byu.edu](mailto:jaudall@byu.edu)

# Supplemental Tables

**Table S1** Silencing or activation of genes as a result of mutation.

| **Gene ID** | **Description** | ***Li_2_* mean** | ***Li_2_* SD** | **WT mean** | **WT SD** |
| --- | --- | --- | --- | --- | --- |
| Gorai.003G034700 | bHLH DNA-binding | 0 | 0 | 438 | 98 |
| Gorai.006G152000 | NAD(P)-binding Rossmann-fold | 0 | 0 | 182 | 36 |
| Gorai.007G233700 | Unknown | 0 | 0 | 177 | 59 |
| Gorai.001G130200 | RmlC-like cupins superfamily protein | 0 | 0 | 155 | 35 |
| Gorai.007G192800 | Unknown | 0 | 0 | 149 | 33 |
| Gorai.009G138400 | Root hair specific 6 | 0 | 0 | 91 | 30 |
| Gorai.006G198200 | Cytochrome P450, family 715 | 0 | 0 | 72 | 6 |
| Gorai.005G257000 | SAUR-like auxin-responsive protein | 0 | 0 | 56 | 16 |
| Gorai.001G113300 | UDP-glucosyl transferase 76E2 | 0 | 0 | 49 | 4 |
| Gorai.009G170700 | NAC domain containing protein | 0 | 0 | 48 | 15 |
| Gorai.002G002600 | SKP1-like 4 | 0 | 0 | 45 | 8 |
| Gorai.002G040300 | Receptor like protein 15 | 0 | 0 | 43 | 8 |
| **Gene ID** | **Description** | ***Li_2_* mean** | ***Li_2_* SD** | **WT mean** | **WT SD** |
| Gorai.002G107900 | ABA glycosyltransferase UGT73C14 | 772 | 66 | 0 | 0 |
| Gorai.007G105600 | Cytokinin response factor 6 | 246 | 56 | 0 | 0 |
| Gorai.009G057500 | Concanavalin A-like lectin protein kinase | 239 | 49 | 0 | 0 |
| Gorai.006G275000 | Protein of unknown function (DUF607) | 232 | 33 | 0 | 0 |
| Gorai.009G300400 | Unknown | 205 | 42 | 0 | 0 |
| Gorai.005G099500 | PHD finger transcription factor | 201 | 46 | 0 | 0 |
| Gorai.010G101500 | Unknown | 187 | 3 | 0 | 0 |
| Gorai.013G163300 | COBRA-like protein 10 precursor | 146 | 6 | 0 | 0 |
| Gorai.012G158100 | Protein of unknown function (DUF241) | 145 | 8 | 0 | 0 |
| Gorai.013G100400 | Cation/H+ exchanger 4 | 140 | 36 | 0 | 0 |
| Gorai.007G329600 | Cysteine proteinases superfamily protein | 138 | 21 | 0 | 0 |
| Gorai.007G195200 | Plant protein of unknown function (DUF827) | 124 | 34 | 0 | 0 |
| Gorai.005G242700 | Polynucleotidyl transferase, ribonuclease H-like | 118 | 18 | 0 | 0 |
| Gorai.006G171300 | Transmembrane amino acid transporter | 117 | 21 | 0 | 0 |
| Gorai.010G120700 | Glutamine dumper 4 | 100 | 33 | 0 | 0 |
| Gorai.001G257400 | P-loop containing nucleoside triphosphate hydrolases | 97 | 27 | 0 | 0 |
| Gorai.009G313800 | Leucine-rich repeat protein kinase | 88 | 8 | 0 | 0 |
| Gorai.011G048600 | Tetratricopeptide repeat (TPR)-like | 88 | 28 | 0 | 0 |
| Gorai.012G132200 | MYB-like 102 | 87 | 8 | 0 | 0 |
| Gorai.005G099200 | Unknown | 87 | 9 | 0 | 0 |
| Gorai.001G209400 | Unknown | 77 | 0 | 0 | 0 |
| Gorai.007G321500 | F-box/RNI-like/FBD-like domains-containing protein | 74 | 9 | 0 | 0 |
| Gorai.002G171300 | ENTH/VHS/GAT family protein | 66 | 16 | 0 | 0 |
| Gorai.010G038800 | F-BOX WITH WD-40 2 | 64 | 10 | 0 | 0 |
| Gorai.004G008600 | Protein of unknown function (DUF567) | 57 | 2 | 0 | 0 |
| Gorai.001G143500 | Cytochrome P450, family 82 | 57 | 4 | 0 | 0 |
| Gorai.008G214500 | F-box family protein | 57 | 4 | 0 | 0 |
| Gorai.009G351500 | Unknown | 56 | 18 | 0 | 0 |
| Gorai.002G105600 | RING/U-box superfamily protein | 54 | 1 | 0 | 0 |
| Gorai.003G093300 | nuclear factor Y, subunit A9 | 52 | 11 | 0 | 0 |
| Gorai.006G215900 | Protein of unknown function (DUF1278) | 51 | 7 | 0 | 0 |
| Gorai.009G050200 | Integrase-type DNA-binding superfamily protein | 49 | 11 | 0 | 0 |
| Gorai.004G154500 | Unknown | 48 | 11 | 0 | 0 |
| Gorai.001G079200 | Protein kinase superfamily protein | 47 | 4 | 0 | 0 |
| Gorai.009G157300 | WRKY DNA-binding protein 28 | 45 | 12 | 0 | 0 |
| Gorai.004G254800 | Unknown | 45 | 13 | 0 | 0 |
| Gorai.007G079000 | Protein of unknown function (DUF679) | 44 | 6 | 0 | 0 |
| Gorai.010G196400 | Protein kinase 2B | 42 | 6 | 0 | 0 |

**Table S2** Mutation effects on functional distribution of homeologs. Fisher’s exact test results.

| **BIN** | **Functional categories** | **A_T_ biased** | ***Li_2_*A/wtA** | **D_T_ biased** | ***Li_2_*D/wtD** | **Fisher’s** |
| --- | --- | --- | --- | --- | --- | --- |
| 1 | Photosynthesis | 2958 | 2 | 1620 | 0 | 0.417639 |
| 2 | Major carbohydrates | 2958 | 2 | 1620 | 0 | 0.417639 |
| 3 | Minor carbohydrates | 2958 | 3 | 1620 | 2 | 0.758818 |
| 4 | Glycolysis | 2958 | 0 | 1620 | 0 | 1 |
| 5 | Fermentation | 2958 | 0 | 1620 | 2 | 0.125371 |
| 6 | Gluconeogenesis | 2958 | 0 | 1620 | 1 | 0.354007 |
| 7 | OPP pathway | 2958 | 0 | 1620 | 1 | 0.354007 |
| 8 | TCA | 2958 | 0 | 1620 | 0 | 1 |
| 9 | ATP synthesis | 2958 | 0 | 1620 | 0 | 1 |
| 10 | Cell wall | 2958 | 10 | 1620 | 12 | 0.051973 |
| 11 | Lipid metabolism | 2958 | 10 | 1620 | 6 | 0.67629 |
| 12 | Nitrogen assimilation | 2958 | 0 | 1620 | 1 | 0.354007 |
| 13 | Amino acid metabolism | 2958 | 3 | 1620 | 2 | 0.758818 |
| 14 | S-assimilation | 2958 | 0 | 1620 | 0 | 1 |
| 15 | Metal handling | 2958 | 0 | 1620 | 2 | 0.125371 |
| 16 | Secondary metabolism | 2958 | 5 | 1620 | 8 | 0.0496 |
| 17 | Hormones | 2958 | 7 | 1620 | 6 | 0.265898 |
| 18 | Cofactor and vitamin synthesis | 2958 | 0 | 1620 | 0 | 1 |
| 19 | Tetrapyrrole synthesis | 2958 | 0 | 1620 | 0 | 1 |
| 20 | Stress | 2958 | 10 | 1620 | 14 | 0.018502 |
| 21 | Redox | 2958 | 2 | 1620 | 0 | 0.417639 |
| 22 | Polyamine synthesis | 2958 | 0 | 1620 | 0 | 1 |
| 23 | Nucleotide metabolism | 2958 | 1 | 1620 | 1 | 0.582644 |
| 24 | Biodegradation of Xenobiotics | 2958 | 1 | 1620 | 1 | 0.582644 |
| 25 | C1-metabolism | 2958 | 0 | 1620 | 0 | 1 |
| 26 | Misc. enzyme families | 2958 | 24 | 1620 | 22 | 0.05884 |
| 27 | RNA | 2958 | 21 | 1620 | 16 | 0.265818 |
| 28 | DNA | 2958 | 2 | 1620 | 3 | 0.241524 |
| 29 | Protein | 2958 | 37 | 1620 | 16 | 0.263117 |
| 30 | Signaling | 2958 | 28 | 1620 | 23 | 0.12666 |
| 31 | Cell | 2958 | 12 | 1620 | 6 | 0.535528 |
| 32 | micro RNA | 2958 | 0 | 1620 | 0 | 1 |
| 33 | Development | 2958 | 9 | 1620 | 11 | 0.057923 |
| 34 | Transport | 2958 | 16 | 1620 | 11 | 0.33618 |
| 35 | Not assigned | 2958 | 77 | 1620 | 58 | 0.053028 |

**Table S3** Primer’s sequences for detection expression of homeolog pairs.

| ***G. raimondii* accession** | **Locus specific primer, 5’-3’** | **A_T_ subgenome specific primer, 5’-3’** | **D_T_ subgenome specific primer, 5’-3’** |
| --- | --- | --- | --- |
| Gorai.002G223800 | cgttagatatttctcattcttgatcggattggtt | cggcttgaccttgttggaaaaccc | cggcttgaccttgtcggaaagtcg |
| Gorai.007G298400 | ccgagcaacatgcatttttgtacaagg | atgaatctggtgttttccaattttgttcaacc | gaatctggtgttttccaattttgttcgtca |
| Gorai.012G107500 | ggagttttgcctaatttctgtgcaaccatatt | cggatgaagaaaggatcagctgggtac | cggatgaagaaaggatcagctggcatt |
| Gorai.006G168500 | gggtcccaaaggattgagctgtta | gcacttggccaatggcctta | gcacttggccaatggccctg |
| Gorai.011G272700 | tttgaagattctgcatttctctgattagatg | acaacaacgatgatgtttgtgttaaggtc | acaacaacgatgatgtttgtgttaaggtt |
| Gorai.001G219000 | gggcaaggcatggaaaagga | actgaacaaggcatttacaattttcgct | caacactgaacaaggcattacaattttctta |
| Gorai.007G025300 | cagaaatcagcagcgtggatgag | ctcccacaagcgacgggagt | ctcccacaagcgacgggatc |
| Gorai.008G281400 | tggtttcctcttcttcttcttcctcg | ccacgacgccgtatcgattagtc | cgacgccgtatcgatttgcg |
| Gorai.007G170600 | ggggtattttgatccataacccactaaat | cctccaagatttgaaccaaaacctaca | tccaagatttgaaccaaaaccttcg |
| Gorai.007G057400 | ggggtaacgggacttgtcggt | atgtccggttccagggccatc | atgtccggttccagggcgagt |
| Gorai.006G195700 | ccttggcaataggtggtccaaaat | tggtttttgtaaaggctcatgtttgg | ggtttttgtaaaggctcatgtggga |
| Gorai.007G056400 | tgtaagccgaggtgcattacagagtaa | ccgattcttgtactcttcccgatacg | catccgattcttgtactcttcccgatata |
| Gorai.007G005700 | ctctgcaacacagtgactattggtagctt | ccaggaagagggaaaagagcaaaata | caggaagagggaaaagagcaaaatg |
| Gorai.007G277000 | tggtgtttgagaaagatcttaaccttgatg | gcaaggctcttttgttactgatcctatg | caaggctcttttgttactgatccgaat |

**Table S4** Primer’s sequences.

| ***G. raimondii* accession** | **Forward primer sequence, 5’-3’** | **Reverse primer sequence, 5’-3’** |
| --- | --- | --- |
| Gorai.003G034700 | CATTAATGAAGCTTCCGACAGACC | GACATGGTTGATGTTTGTGATGGT |
| Gorai.007G233700 | TGGCATATCAGAATCTGGGCATAA | TATCTTGGTTCCACCCTCCATTTT |
| Gorai.001G130200 | TTCCAGTTTGATCCTTTTCGTTCG | CACATCCCCTATCTTCAATCGTCT |
| Gorai.007G192800 | CACCCTGTTAGGATCAGAAATCCA | CGAATCCAAGGTATCGGATAGAGG |
| Gorai.007G105600 | CTTGATCCCCATGGTAGATATCGG | ACAGCTCGATCAATTGTTTTCTGG |
| Gorai.009G057500 | GTCAAGAATCTCAGTCCCTCTGTT | CATCTGATAAGCTGGTTGGTTGTC |
| Gorai.006G275000 | CTAATGTCTCTGGGGATGGTATGG | GCATCTGGATCCCATTCATTCTTG |
| Gorai.005G099500 | AAAATGCTCAACAAACCACAAACG | GTAAACTCTCTCGCATCTCCATCT |
